# Three-Dimensional Assessment of the Condylar Position in Different Malocclusions Using Cone-Beam Computed Tomography: A Cross-Sectional Study

**DOI:** 10.7759/cureus.75704

**Published:** 2024-12-14

**Authors:** Altaf H Thekiya, Ayesha Mahmood, Zuber A Naqvi, Danish Uz Zama Khan, Shilpa Pharande, Manish Sharma, Seema Gupta

**Affiliations:** 1 Department of Orthodontics, Diamond Dental Care, Nanded, IND; 2 Department of Orthodontics, Sathya's Dental Zone, Hyderabad, IND; 3 Department of Orthodontics, Vishwas Dental Care, Jaipur, IND; 4 Department of Dentistry, Era’s Lucknow Medical College and Hospital, Lucknow, IND; 5 Department of Orthodontics, Sinhgad Dental College and Hospital, Pune, IND; 6 Department of Oral Pathology, Jawahar Medical Foundations Annasaheb Chudaman Patil Dental College, Dhule, IND; 7 Department of Orthodontics, Kothiwal Dental College and Research Centre, Moradabad, IND

**Keywords:** condylar, cone-beam computed tomography, position, skeletal, temporomandibular joint

## Abstract

Introduction

The role of the condylar position in the correct functioning of the stomatognathic system has been the center of the study. Using cone-beam computed tomography (CBCT), this study looked at the three-dimensional (3D) position of the condylar bone in patients from Class I, Class II, Division 1, and Division 2.

Materials and methods

This cross-sectional, retrospective study was conducted using 102 CBCT records, with 34 records allocated to each category of malocclusion classification, such as dentoskeletal Class I, skeletal Class II, and dental Class II, Division 1 and 2. CBCT scans were conducted utilizing a Carestream New Generation CBCT apparatus (Carestream Dental, Atlanta, Georgia) in accordance with a standardized protocol (operating at a voltage of 120 kV, a current of 80 mA, a seven-second scan time, a field of view (FOV) measuring 10 x 10 cm, and a resolution of 0.2 voxels, and 1-mm slice thickness). The condylar position was assessed as the superior, inferior, and medial distance of the condyle from the glenoid fossa, along with the condylar angle. The distance from the most anterior point on the anterior surface of the condyle to the articular eminence was taken as anterior condylar distance; the distance of the superior surface of the condyle from the deepest point of the glenoid fossa was taken as superior condylar distance; the distance of the posterior surface of the condyle from the glenoid fossa was taken as posterior condylar distance; the condylar angle was measured as an angle between the XY line and the FH' line passing through X, where X is the center of the condyle; and the distance of the medial surface of the condyle from the glenoid fossa was taken as medial distance. The data were then subjected to statistical analyses.

Results

For anterior distance, the highest distance was noted in Class II Division 1 (3.32 ± 0.4 mm), and the lowest was seen in Class I (2.43 ± 0.26 mm). In the posterior distance, Class I exhibited the highest mean distance of 2.05 ± 0.14 mm, while Class II Division 1 showed the lowest distance of 1.83 ± 0.18 mm. For superior distance, the highest mean value was noticed in Class I patients at 2.92 ± 0.22 mm, and the lowest value was seen in Class II Division 1 at 2.61 ± 0.35 mm (p=0.001). For the condylar angle, the highest mean value was observed in Class I (30.96 ± 1.91^0^) and the lowest in Class II Division 1 (26.71 ± 1.48^0^), with p=0.001. Confirmatory factor analysis revealed that the most substantial loading was attributed to the condylar angle at -2.28, signifying its significant contribution to Fc1.

Conclusion

The condyle was placed anteriorly, superiorly, and medially in Class II Division 1 and posteriorly in Class II Division 2, compared to Class I patients.

## Introduction

The significance of condylar positioning in ensuring the proper operation of the stomatognathic system has been a focal point of investigation and debate throughout the evolution of dental science. Existing scholarly work encompasses numerous publications that aim to ascertain whether condylar concentricity represents the ideal position and whether any deviations from this alignment could play a pivotal role in the onset of temporomandibular joint (TMJ) disorders [1,2].

Class II Division 2 patients exhibit posterior displacement of the mandible, which becomes entrapped within the glenoid fossa. This hypothesis is further substantiated by subjective clinical observations, which suggest that retroclined maxillary incisors may serve as significant occlusal interference in patients who present with an absence of overjet [3]. However, controversial results were reported in a study in which no significant differences were observed between Class I and Class II Division 2 patients [4].

In a study by Arieta-Miranda et al. [5], superior positioning of the condyle was observed in Class II patients than in Class I. Class II Division 1 exhibited reduced measurements for the anterior distance, indicating a more anteriorly positioned condyle, whereas the Class II Division 2 cohort displayed a more posteriorly [2]. 

The emergence of three-dimensional diagnostic modalities has facilitated the procurement of substantially enhanced imaging outcomes. The introduction of cone-beam computed tomography (CBCT), which delivers high-resolution three-dimensional (3D) visualizations that aid in the evaluation and quantification of facial osseous structures in their true dimensions (1:1 scale) without appreciable magnification or distortion, thereby affording improved anatomical accuracy [6]. Therefore, the present study aimed to evaluate the 3D condylar position in Class I, Class II, Division 1, and Division 2 patients using cone-beam computed tomography (CBCT). The null hypothesis posited for this study was that there would be no significant differences in the condylar position between the groups.

## Materials and methods

Study design and setting

This cross-sectional, retrospective study was conducted using CBCT records of skeletal Class I, skeletal Class II with dental Class II, Division 1 and Division 2 patients who visited the Department of Orthodontics, Kothiwal Dental College and Research Centre, Moradabad, India, between January 2019 and December 2023. This study was approved by the Institutional Ethics Committee Review Board (KDCRC/IERB/04/2024/52) and followed the principles of the Declaration of Helsinki. Written informed consent was obtained from all patients as a routine procedure to use their records for research purposes while maintaining confidentiality.

Eligibility criteria

Those with complete records of dentoskeletal Class I (angle between point A, B as the deepest point in the anterior concavity of the maxilla and mandible and nasion (N) as the most anterior point on the frontonasal suture as the ANB angle of 2-4^0^), skeletal Class II patients with dental Class II, Division 1 and 2 patients with an average growth pattern (ANB > 4^0^, mandibular plane angle as SN-GoGn <27^0^), age > 20 years, irrespective of any gender, absence of any TMJ abnormality, and no degenerative diseases of the condyle, were included in the present study. Patients with incomplete records, a history of previous orthodontic treatment, TMJ trauma, mandibular asymmetry, skeletal Class III, congenital abnormalities such as cleft lip and palate or other craniofacial anomalies, and those requiring orthodontic camouflage were excluded from the study. 

Sample size estimation

G Power software version 3.2.9 (Heinrich-Heine-Universität Düsseldorf, Düsseldorf, Germany) was used for sample size estimation. A minimum of 102 total samples were estimated considering an effect size of 0.51 for the anterior distance of the mandibular condyle in the TMJ [2]. The power of the study was 80% with an alpha error of 5%.

Methodology

CBCT scans in the records were conducted using a Carestream New Generation CBCT apparatus (Carestream Dental, Atlanta, Georgia) in accordance with a standardized protocol (operating at a voltage of 120 kV, a current of 80 mA, a seven-second scan time, a field of view (FOV) measuring 10 × 10 cm, a resolution of 0.2 voxels, and 1-mm slice thickness). To achieve consistent head orientation, the subjects were positioned supine with the application of a head stabilizer. During CBCT scanning, patients were given specific instructions to maintain an erect seated posture with the teeth on maximum intercuspation and to not swallow. The eyelids and nasal dorsum were employed as the horizontal and vertical reference axes, respectively, and were established using a laser beam. Image reconstruction for visual analysis was performed using the CS imaging software (CS Imaging Software Version 8, Carestream Dental, Atlanta, Georgia, USA). The reference planes and points used in the study are listed in Table 1.

**Table 1:** Landmarks and planes used in the study.

Reference points and planes	Definition of points and planes
X point	Center of condyle
A point	The most anterior point on condyle
A’ point	The nearest point to A point on glenoid fossa
Anterior distance (mm)	The distance of condyle from glenoid fossa in anterior direction as represented by AA’ line
B point	The most superior point on condyle
B’ point	The nearest point to B point on glenoid fossa
Superior distance (mm)	The distance of condyle from glenoid fossa in superior direction as represented by BB’ line
C point	The most posterior point on condyle
C’ point	The nearest point to C point on glenoid fossa
Posterior distance (mm)	The distance of condyle from glenoid fossa in posterior direction as represented by CC’ line
D point	The most medial point on condyle
D’ point	The nearest point to D point on glenoid fossa
Medial distance (mm)	The distance of condyle from glenoid fossa in medial direction as represented by DD’ line
FH plane	A line passing through uppermost point of external auditory meatus (Y) and lowermost point of bony orbit (Or)
FH’ line	A line parallel to FH plane passing through X point
Condylar angle (^0^)	Angle between FH’ line and XY line

**Figure 1:**
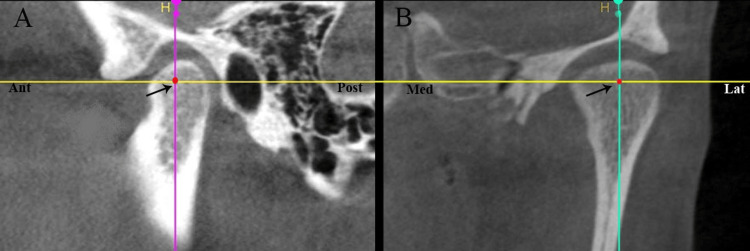
Assessment of the centre of the condyle. A: in anteroposterior plane; B: in mediolateral plane

The 3D assessment of the center of the condyle in sagittal and coronal plane has been represented in Figure 1.

**Figure 2:**
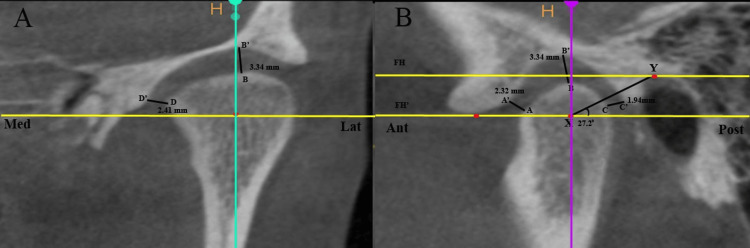
Measurements of condylar position in mediolateral and anteroposterior plane. AA’: distance of anterior surface of condyle from the articular eminence; BB’: distance of superior surface of condyle from the deepest point of glenoid fossa; CC’: distance of posterior surface of condyle from glenoid fossa; condylar angle: angle between XY line and FH' line passing through X, where X is the center of the condyle; DD’: distance of medial surface of condyle from the glenoid fossa.

The distance from the most anterior point on the anterior surface of the condyle to the articular eminence was taken as anterior condylar distance; the distance of the superior surface of the condyle from the deepest point of the glenoid fossa was taken as superior condylar distance; the distance of the posterior surface of the condyle from the glenoid fossa was taken as posterior condylar distance; the condylar angle was measured as an angle between the XY line and the FH' line passing through X, where X is the center of the condyle; and the distance of the medial surface of the condyle from the glenoid fossa was taken as medial distance. The assessment of condylar position in the anterior, posterior, superior, and medial directions, and condylar angle, are shown in Figure 2.

CBCT scans were evaluated by two trained examiners (DUZK, SP) who were blinded to the group allocation. The evaluators were briefed about the procedure of measurements, and the measurements were repeated at two-week intervals on 20 randomly selected CBCTs. The interclass correlation coefficient value of 0.90 and the intraclass correlation coefficient value of 0.94 showed high reliability and reproducibility.

Statistical analysis

The statistical analysis was performed by a statistician (MS) who was being provided with the coded data and therefore was blinded to group allocation. The collected measurements were systematically entered into a Microsoft Excel spreadsheet (Microsoft Corporation, Redmond, Washington, United States) and subsequently analyzed using IBM SPSS Statistics for Windows, Version 23 (Released 2015; IBM Corp., Armonk, New York, United States). Descriptive statistics, including frequency distributions, means, and standard deviations (SD), were used to summarize the data. Study groups consisting of Class 1, Class II Division 1, and Class II Division 2 malocclusion patients were compared using two-way analysis of variance (ANOVA). Statistical significance was set at a threshold of p<0.05. Additionally, confirmatory factor analysis (CFA) was conducted to identify the variable that most strongly influenced the position of the mandibular condyle within the TMJ.

## Results

The study encompassed 102 records, with 34 records allocated to each category of malocclusion classification and gender. Class I males exhibited a mean age of 24.89 ± 2.54 years, Class II Division 1 revealed a mean age of 24.82 ± 2.82 years, and Class II Division 2 indicated a mean age of 25.09 ± 2.56 years. In contrast, the females displayed greater variability (Table 2).

**Table 2:** Descriptive statistics of the study sample. Data represented in form of n (%), and mean and standard deviation.

Groups	Parameters	Frequency (n)	%	Mean age in years	Standard deviation
Class I	Male	18	17.65%	24.89	2.54
	Female	16	15.69%	27.5	4.07
Class II Division 1	Male	22	21.57%	24.82	2.82
	Female	12	11.76%	23.17	1.75
Class II Division 2	Male	22	21.57%	25.09	2.56
	Female	12	11.76%	23.83	2.86

The null hypothesis was rejected, as significant differences were observed between the groups for all measured variables. For anterior distance, the highest distance was noted in Class II Division 1 (3.32 ± 0.4 mm), and the lowest was seen in Class I (2.43 ± 0.26 mm). Significant differences were observed between all group comparisons in the post-hoc analysis. In posterior distance, Class I exhibited the highest mean distance of 2.05 ± 0.14 mm, while Class II Division 1 showed the lowest distance of 1.83 ± 0.18 mm. The differences were statistically significant (p=0.001), with significant post hoc differences between Class I and Class II Division 1, and Class II Division 1 and Class II Division 2. For superior distance, the highest mean value was noticed in Class I patients of 2.92 ± 0.22 mm, and the lowest value was seen in Class II Division 1 of 2.61 ± 0.35 mm, with p=0.001. Significant differences were observed between Class I and Class II Division 1 and Class II Division 2 and Class I. In medial distance, Class I showed the highest mean value of 2.19 ± 0.27 mm, while Class II Division 1 showed the lowest value of 1.98 ± 0.21 mm, with p=0.001. Post-hoc analysis revealed significant differences between Class I and Class II Division 1. This showed that the condyle was placed anteriorly, superiorly, and medially in Class II Division 1 and posteriorly in Class II Division 2 compared with Class I patients. For the condylar angle, the highest mean value was observed in Class I (30.96 ± 1.91^0^) and the lowest in Class II Division 1 (26.71 ± 1.48^0^), with p=0.001. Significant differences were observed between the groups (Table 3).

**Table 3:** Comparative analysis of condylar position between the groups. p < 0.05: significant (S); CI: confidence interval; LL: lower limit; UL: upper limit; ANOVA: analysis of variance; p < 0.05: significant (S); NS: non-significant Data presented in form of mean ± standard deviation (SD)

Parameters	Groups	95% CI (LL-UL)	Mean ± SD	F-value using ANOVA	p-value	Post-hoc analysis using Tukey test
Age in years	Class I	24.88 - 27.36	26.12 ± 3.55	3.76	0.027*	Class I vs. Class II Division 1 (S)
	Class II Division 1	23.33 - 25.14	24.24 ± 2.59			Class II Division 1 vs. Class II Division 2 (NS)
	Class II Division 2	23.71 - 25.59	24.65 ± 2.72			Class 2 Division II vs. Class 1 (NS)
Anterior condylar distance in mm	Class I	2.34 - 2.53	2.43 ± 0.26	51.57	0.001*	Class 1 vs. Class II Division 1 (S)
	Class II Division 1	3.18 - 3.46	3.32 ± 0.41			Class II Division 1 vs. Class II Division 2 (S)
	Class II Division 2	2.84 - 3.13	2.98 ± 0.41			Class 2 Division II vs. Class 1 (S)
Posterior condylar distance in mm	Class I	2.01 - 2.12	2.05 ± 0.14	19.12	0.001*	Class I vs. Class II Division 1 (S)
	Class II Division 1	1.77 - 1.93	1.83 ± 0.18			Class II Division 1 vs. Class II Division 2 (S)
	Class II Division 2	1.96 - 2.06	2.01 ± 0.15			Class II Division 2 vs. Class I (NS)
Superior condylar distance in mm	Class I	2.84 – 3.04	2.92 ± 0.22	8.19	0.001*	Class I vs. Class II Division 1 (S)`
	Class II Division 1	2.48 - 2.73	2.61 ± 0.35			Class II Division 1 vs. Class II Division 2 (NS)
	Class II Division 2	2.58 - 2.85	2.71 ± 0.38			Class II Division 2 vs. Class I (S)
Medial condylar distance in mm	Class I	2.09 - 2.28	2.19 ± 0.27	7.2	0.001*	Class I vs. Class II Division 1 (S)
	Class II Division 1	1.91 - 2.05	1.98 ± 0.21			Class II Division 1 vs. Class II Division 2 (NS)
	Class II Division 2	2.05 - 2.18	2.11 ± 0.19			Class II Division 2 vs. Class I (NS)
Condylar angle in degrees	Class I	30.31 - 31.63	30.96 ± 1.91	62.31	0.001*	Class I vs. Class II Division 1 (S)
	Class II Division 1	26.19 - 27.23	26.71 ± 1.48			Class II Division 1 vs. Class II Division 2 (S)
	Class II Division 2	27.75 - 28.67	28.21 ± 1.33			Class II Division 2 vs. Class I (S)

**Figure 3:**
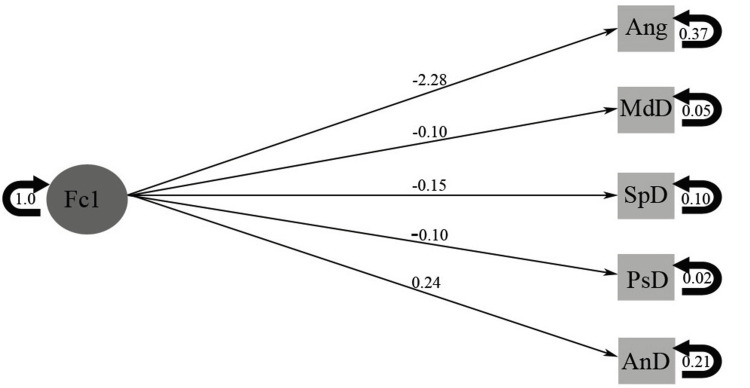
Confirmatory factor analysis. Fc1: condyle position; Ang: condylar angle; MdD: medial distance; AnD: anterior distance; SpD: superior distance; PsD: posterior distance

The CFA model delineated the interrelationship between the latent construct Fc1 (condyle position) and five observable variables: condylar angle (Ang), medial distance (MdD), anterior distance (AnD), superior distance (SpD), and posterior distance (PsD). Fc1 is a standardized latent factor constrained to a variance of 1. The most substantial loading was attributed to Ang (angle) at -2.28, signifying its significant contribution to Fc1. Other variables showed fewer yet significant associations. Measurement errors for the observable variables are denoted by circular arrows, with corresponding variances such as 0.37 for Ang and 0.21 for AnD. Peak loading was observed for Ang (-2.28), indicating a robust association between the angle variable and positional alterations of the condyle (Figure 3).

## Discussion

The outcomes of this investigation offer substantial contributions to the understanding of positional discrepancies in the mandibular condyle across various malocclusion categories and gender. By examining anterior, posterior, superior, and medial measurements alongside condylar angulations, this study clarified the unique trends in condylar positioning linked to Class I, Class II Division 1, and Class II Division 2 malocclusions. Furthermore, the utilization of a CFA framework bolsters the interpretation of how these variables collectively affect the latent construct Fc1, which signifies the condylar position.

Anterior condylar distance

Class II Division 1 malocclusion exhibited the most pronounced anterior distance, markedly exceeding that of the other classifications. This anterior condylar positioning aligns with research underscoring the forward mandibular orientation frequently observed in Class II Division 1 malocclusions [2,7]. Anterior condyle placement in these individuals may lead to diminished TMJ stability and functional difficulties. The anterior condylar placement in Class II Division 1 individuals might have a compensatory adaptation to a large overjet, which is known as a "Sunday bite." Conversely, Class I malocclusion, characterized by the least anterior distance, corresponds to the normative condylar positioning typical of balanced occlusion [8].

Posterior distance

The positioning of the posterior condyles was notably greater in individuals with Class I malocclusion (2.05 ± 0.14 mm) than in those exhibiting Class II Division 1 malocclusion (1.83 ± 0.18 mm). The statistically significant disparities observed between these classifications underscore the complex relationship between the posterior condylar positioning and occlusal stability. Posterior positioning in patients classified as Class I may indicate a predisposed centric relationship [2]. Conversely, Class II Division 2 malocclusions, characterized by retroclination of the incisors, may demonstrate unique posterior condylar configurations resulting from altered mandibular dynamics [2,9,10]. These individuals typically exhibit a backward path of mandibular closure, culminating in a posterior displacement of the mandibular condyle, which may precipitate undue stress on the retrodiscal tissues and TMJ pathologies in these subjects. Consequently, in such cases, it is imperative to rectify the retroclination of incisors through orthodontic intervention, thereby establishing an appropriate overjet, ameliorating the backward path of closure, and consequently, rectifying the posterior condylar positioning.

Superior distance

The superior positioning of the condyle in Class I malocclusion (2.92 ± 0.22 mm) and inferior in Class II Division 1 (2.61 ± 0.35 mm) implies a direct association between occlusal and skeletal equilibrium. This superior displacement may reflect adaptation to functional stresses aligned with the biomechanical frameworks of condylar remodeling [11]. The diminished superior alignment in Class II Division 1 could render these individuals susceptible to altered TMJ biomechanics, potentially precipitating dysfunction over time [12]. As a result of the substantial overjet, individuals classified as Class II Division 1 tend to position their mandible in an anterior and inferior manner as a compensatory adaptation. Consequently, it is imperative to rectify the excessive overjet in these individuals to ameliorate the aberrant condylar positioning.

Medial distance

The positioning of the medial condyle exhibited statistically significant variances among the different classes of malocclusion, with Class I subjects presenting the highest measurements and Class II Division 1 subjects exhibiting the lowest measurements [13]. This medial displacement observed in Class II Division 1 may indicate structural modifications in response to anterior mandibular positioning, thereby supporting conclusions drawn from morphometric studies conducted on analogous populations [14]. Conversely, Class II Division 2 malocclusions display intermediate values, implying more intricate condylar adaptations within this specific subgroup [15].

Condylar angle

The condylar angle exhibited the highest degree of prominence in individuals with Class I malocclusion (30.96 ± 1.91^0^) and was notably diminished in those classified under Class II Division 1 (26.71 ± 1.48^0^) [16]. The substantial loading of the condylar angle (-2.28) within the CFA model further accentuates its pivotal function in assessing the true condylar position. The diminished condylar angles observed in Class II Division 1 malocclusions may reflect a physiological adaptation to anterior mandibular displacement, whereas the augmented condylar angle in Class I malocclusions may be attributable to superior positioning of the condyle in skeletal Class I patients.

Interpretations of the confirmatory factor analysis model

The substantial loading of the condylar angle (-2.28) signifies its crucial function in influencing the positional alterations of the condyle. Additional parameters, including the anterior and superior distances, also demonstrated notable correlations, albeit to a lesser extent. These observations are consistent with biomechanical models that conceptualize the condyle as a dynamic structure subject to the influence of occlusal forces, skeletal morphology, and joint mechanics [17].

Clinical implications

These results hold considerable significance for the diagnosis and treatment of orthodontics. Identifying the positional discrepancies of the condyle in relation to various classes of malocclusion can facilitate customization of therapeutic approaches aimed at restoring optimal temporomandibular joint function. For example, individuals with Class II Division 1 malocclusions may achieve improvements from interventions focused on superior stabilization of the condyle, whereas cases classified as Class II Division 2, characterized by a posteriorly positioned condyle, might necessitate the correction of a deep bite and repositioning of the condyle to a more advantageous location.

Limitations

The retrospective design of the study may have led to a selection bias and affected the generalizability of the results. Furthermore, the study was conducted on average growers; therefore, the effect of condylar position on growth pattern could not be assessed. The present study also did not include skeletal Class III malocclusion because of the lack of a sufficient sample. Additionally, incorporating advanced imaging modalities such as magnetic resonance imaging (MRI) can enhance the precision of condylar measurements and provide insights into soft tissue contributions. Finally, investigating the influence of other factors such as occlusal forces, parafunctional habits, and systemic conditions on condylar positioning could offer a more holistic understanding. Future prospective studies with larger sample sizes are required to address the limitations of this study.

## Conclusions

This study highlights significant condylar positional variations among Class I, Class II Division 1, and Class II Division 2 malocclusions. The anterior, posterior, superior, and medial distances of the condyle, along with the condylar angle, differed significantly across the classifications. Class II Division 1 demonstrated the most anterior, inferior, and medial positioning; Class I showed the most stable alignment; and Class II Division 2 showed posterior positioning of the condyle. These findings underscore the importance of customized orthodontic interventions targeting specific malocclusion-related condylar patterns. Additionally, the CFA model confirmed the critical role of the condylar angle in defining positional shifts, thereby offering valuable insights into diagnosis and treatment planning in orthodontics.
